# Application and evaluation of artificial intelligence 3D preoperative planning software in developmental dysplasia of the hip

**DOI:** 10.1186/s13018-024-04588-0

**Published:** 2024-03-08

**Authors:** Hongbin Xie, Jiafeng Yi, Yijian Huang, Renwen Guo, Yubo Liu, Xiangpeng Kong, Wei Chai

**Affiliations:** 1 School of Medicine, Nankai University, Tianjin, 300071 China; 2 Senior Department of Orthopedics, The Fourth Medical Center of PLA General Hospital, Beijing, 100048 China; 3 National Clinical Research Center for Orthopedics, Sports Medicine and Rehabilitation, Beijing, 100853 China; 4 Medical School of Chinese PLA, Beijing, 100853 China

**Keywords:** Total hip arthroplasty, Preoperative planning, Artificial intelligence, Acetabular cup, Hip dysplasia, Accuracy

## Abstract

**Background:**

Accurate preoperative planning is crucial for successful total hip arthroplasty (THA) for developmental dysplasia of the hip (DDH). The aim of this study was to compare the accuracy of an artificial intelligence-assisted three-dimensional (3D) planning system (AIHIP) with two-dimensional templates in predicting acetabular cup size in THA for DDH.

**Method:**

This study retrospectively analyzed image data from 103 DDH patients who had THA between May 2019 and August 2023. AIHIP was used for 3D planning, and two-dimensional (2D) templates were used by two experienced surgeons. Accuracy was assessed by comparing predicted and actual cup sizes, and potential factors affecting accuracy were analyzed, including gender, side, BMI, and dysplasia classification.

**Results:**

AIHIP had higher accuracy in predicting the acetabular cup size compared to the 2D template. Within ± 0 size, AIHIP’s accuracy was 84.1%, while the 2D template’s was 64.0% (*p* < 0.05). Within ± 1 size, AIHIP's accuracy was 95.1%, while the 2D template’s was 81.1% (*p* < 0.05). Accuracy was unaffected by gender, side, or BMI but was by DDH classification. In subgroup analysis, AIHIP's mean absolute error (0.21 ± 0.54) was significantly lower than the 2D template’s (0.62 ± 0.95) for Crowe II and Crowe III (*p* < 0.05).

**Conclusion:**

AIHIP is superior to 2D templates in predicting the acetabular cup size accurately for THA in DDH patients. AIHIP may be especially beneficial for Crowe II and III DDH patients, as 2D templates may not accurately predict cup size in these cases.

## Introduction

Hip arthroplasty is one of the most effective treatments for end-stage hip disease [1–3]. In patients with developmental dysplasia of the hip (DDH), congenital anomalies lead to changes in the size and shape of the acetabulum. This alteration results in inadequate depth and size of the acetabulum to properly accommodate the prosthesis, thus increasing the difficulty and complexity of the procedure [4]. Therefore, in total hip arthroplasty (THA), it is very important to select the appropriate prosthesis and accurate cup size for patients with DDH. The selection of an appropriate acetabular cup size can increase the fixation of the prosthesis and reduce the risk of prosthesis wear and postoperative dislocation [5]. At present, the traditional preoperative planning method for THA is still to measure the length of the lower limb and hip joint offset on conventional film. Although the two-dimensional template is simple to operate, it is affected by the position and magnification of X-ray projection [6–9]. The accuracy of three-dimensional (3D) preoperative planning is significantly better than that of two-dimensional (2D) planning [10], but 3D software requires a longer training time, complicated operation steps and manual segmentation of hip CT images, which is more complicated than that of two-dimensional preoperative planning software [11, 12].

Artificial intelligence (AI) is an emerging technology that can assist medical staff in organizing data, identifying sizes, and guiding practice. The AI software that we employed, named AIHIP (Beijing Changmugu Medical Technology Co., Ltd., China), is a CT-based artificial intelligence preoperative planning software that has been used in general total hip arthroplasty [6, 11, 12]. However, for DDH, its application value is not clear.

In this study, we retrospectively analyzed the clinical data of patients with DDH who underwent THA in our hospital and compared the accuracy of primary THA cup size using AIHIP and 2D digital templates for preoperative planning of preoperative radiographic data. In addition, we analyzed factors that affected preoperative accuracy, including patient gender, side, BMI, and classification of hip dysplasia. The results of this study are helpful to evaluate the application prospects of AIHIP in patients with DDH.

## Materials and methods

This was a retrospective clinical study to compare the accuracy of preoperative planning for primary THA cup size between the AIHIP and 2D template for DDH patients. The factors influencing the accuracy of the method were analyzed. This study report complies with the STROBE guidelines [13]. This study was approved by the Ethics Committee of our hospital (EC No.: S2019-052–01). All investigations were conducted in accordance with the principles of research ethics. As this was a retrospective study and all patient information was deidentified prior to analysis, informed consent was not needed.

### Study population

Retrospective analysis of 103 DDH patients who had primary THA between May 2019 and August 2023 was conducted. A Pinnacle cup (DePuy, Warsaw, IN, USA) was used in all hips, with excellent postoperative imaging results (40° ± 5° external rotation and 20° ± 5° anterior inclination). Exclusion criteria included history of hip surgery, other diseases affecting hip function, and incomplete medical records or imaging. Cohort comprised 87 women and 16 men, with recorded demographics (age, height, weight, BMI, and hip dysplasia classification).

### Study design

Preoperative planning was performed on all patients with DDH who underwent primary THA with cementless prostheses between May 2019 and August 2023. All patients underwent CT scans and conventional X-ray examinations before the operation. CT scans and X-rays were performed preoperatively, with CT images stored in DICOM format. Intraoperative results, including cup size, were recorded and compared to postoperative radiographic measurements of AIHIP and 2D templates. Two senior orthopedic surgeons performed all 2D template preoperative planning and radiographic measurements, with results reviewed and approved by a senior chief surgeon. The images were anonymized, and physicians were blinded to intraoperative results. Intraobserver repeatability was verified by repeating measurements after 2 weeks.

All THAs were performed by the same senior chief arthroplasty surgeon. The same posterolateral approach was used. The cup size was determined by the surgeon during surgery and obtained from the operative notes.

### Preoperative planning using AIHIP software

AIHIP software was used for preoperative planning. Standard anteroposterior X-ray of the pelvis and 256-slice CT plain scan of both hips were taken preoperatively. The scanning range was the whole pelvis and 15 cm below the lesser trochanter of the femur, and the scanning slice thickness was 0.8 mm.

**Fig. 1:**
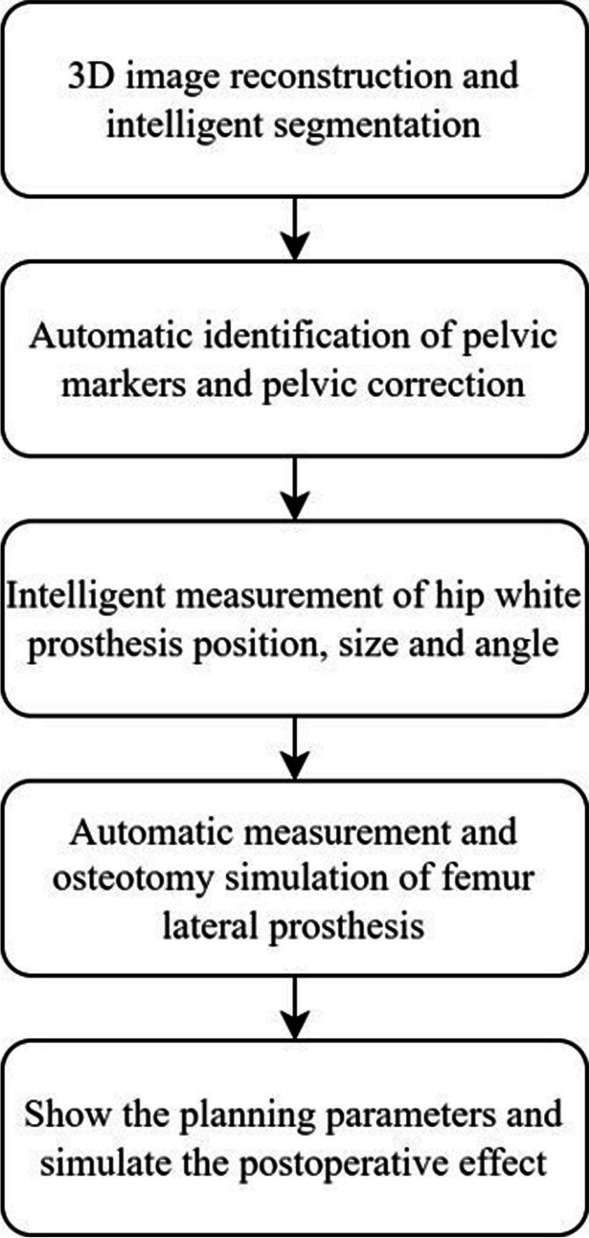
Flow chart of the preoperative AIHIP system planning

CT data for each patient were input into AIHIP software, which segmented and recognized the pelvis and femur using pattern recognition technology. Key points on bones, such as the lesser trochanter and anterior superior iliac spine, were accurately located using a neural network. An automated search engine based on a database and deep learning was then used to match the best prosthesis and plan for optimal outcomes (Fig. 1).

**Fig. 2:**
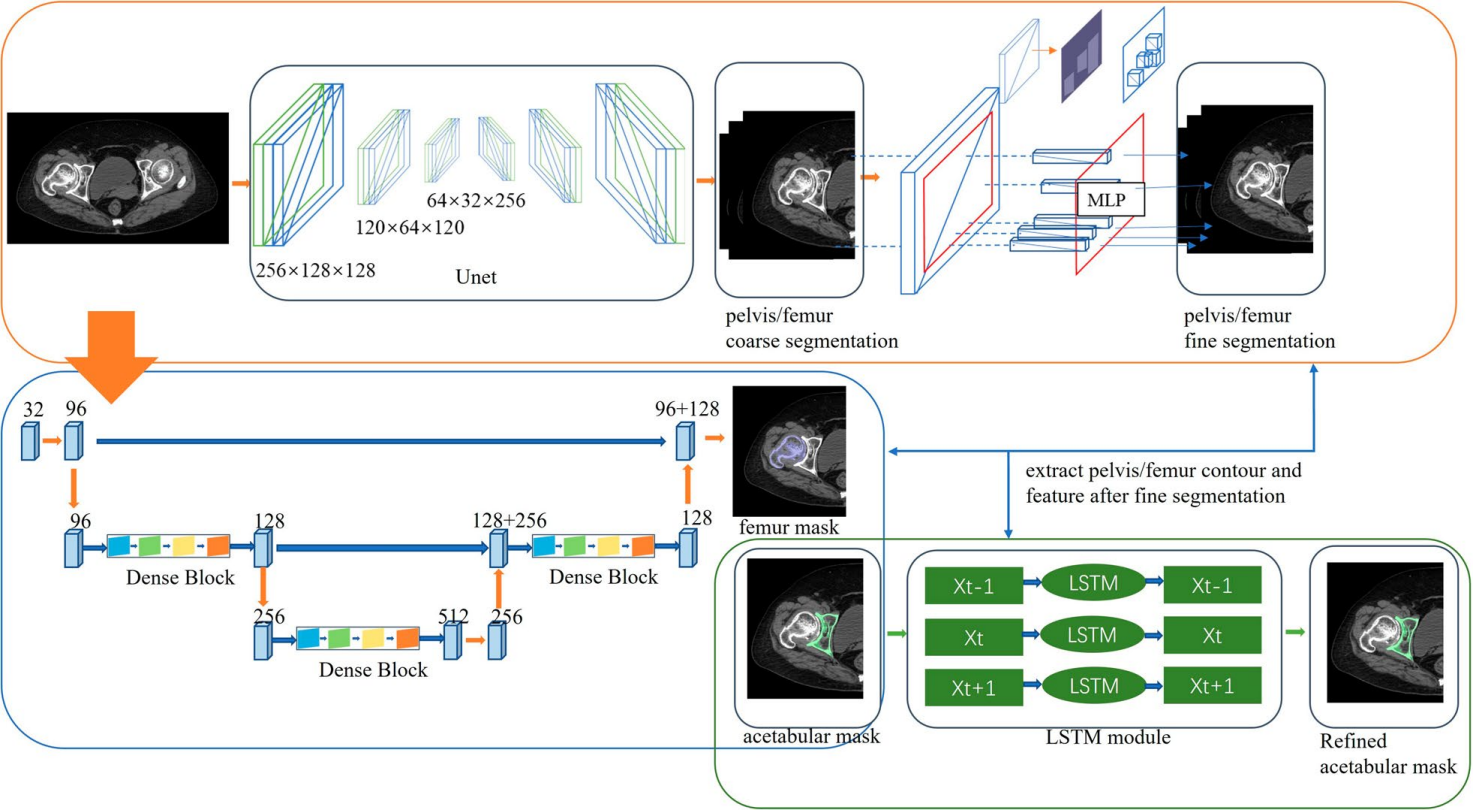
Schematic diagram of bone segmentation based on the unique algorithm (G-NET neural network) developed by the AIHIP system

**Fig. 3:**
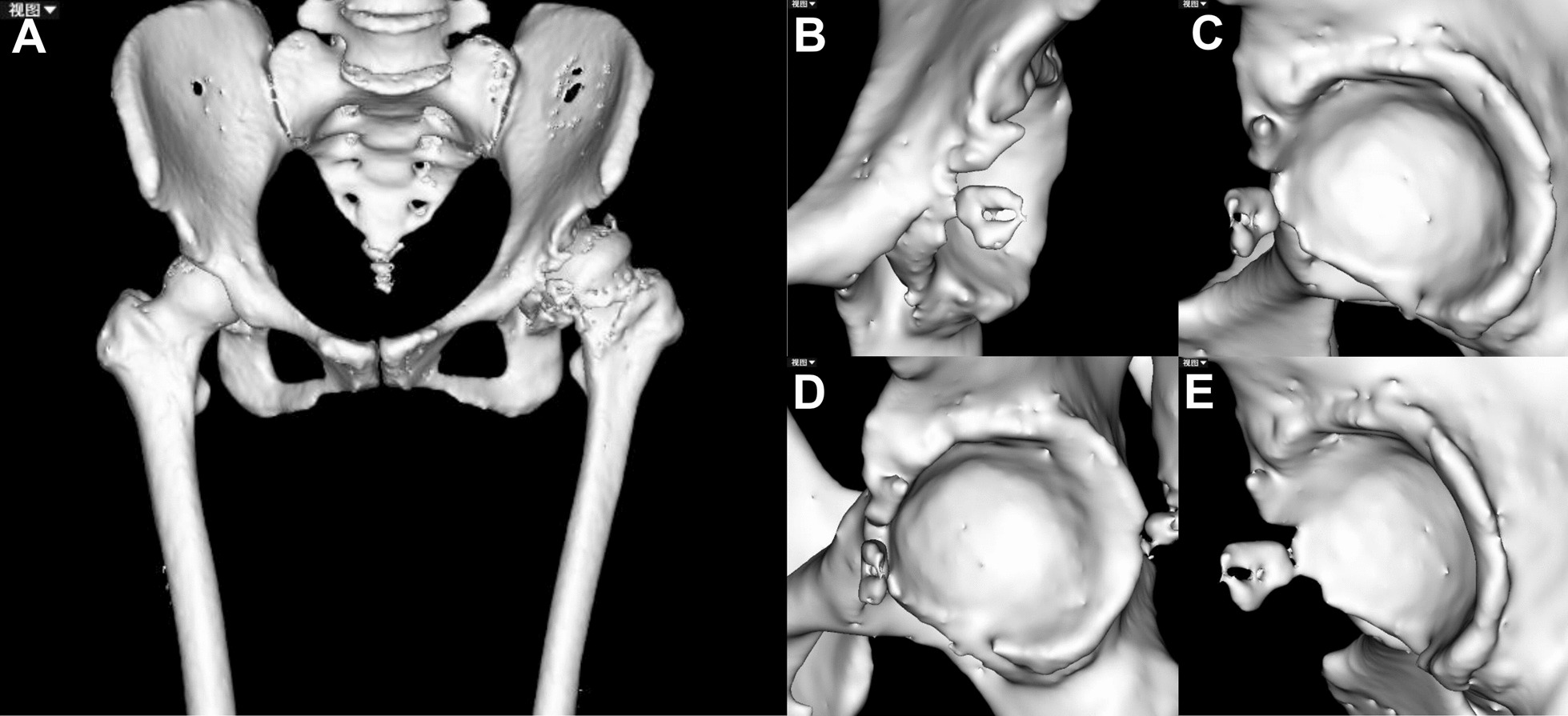
Schematic diagram of AIHIP. **A** 3D reconstruction of the hip joint; **B**–**E** Acetabular morphology from different perspectives

**Fig. 4:**
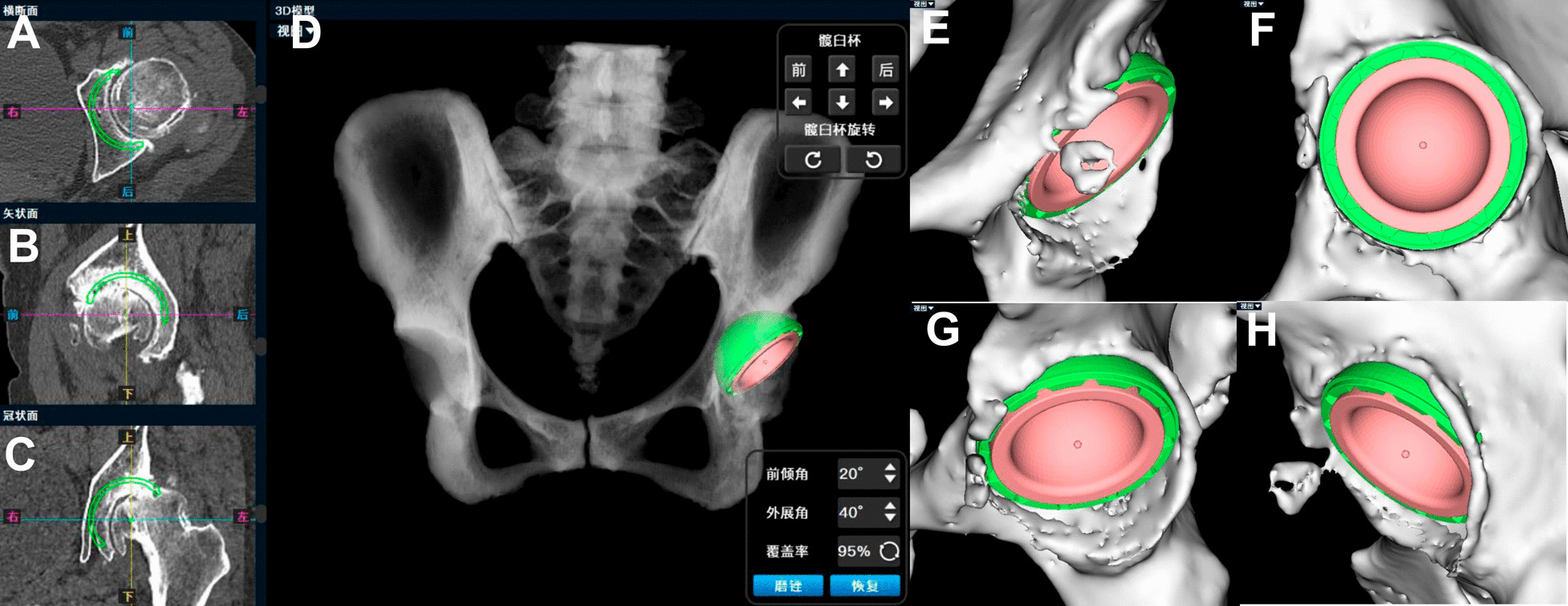
Schematic diagram of placement of the AI hip joint simulation cup position. **A**–**C** Software work interface, simulating cup position. **D** 3D simulation diagram of acetabular cup position. **E**–**H** Acetabular cup from different perspectives

The AI planning converts hip CT data from DICOM to “cmg” format, which is imported into the AIHIP software (Changmugu). The software uses AI to automatically segment the bones and create a 3D model of the pelvis and femur. (Fig. 2). By enlarging or rotating the 3D image, the severity of the lesion can be determined by visualizing the 3D image (Fig. 3). The neural network automatically corrects the pelvis and simulates and calculates the pelvis anteroposterior X-ray, femoral offset, and unequal lower limb length. This software offers both 2D and 3D views, enables real-time calculation of coverage rates, and also simulates postoperative pelvic radiographs (Fig. 4).

For Crowe I and IV DDH patients, the AIHIP places the acetabular cup anatomically. However, for Crowe II and III DDH patients, the templating steps are as follows: (1) In the 2D view of AIHIP, the acetabular cup is automatically set at a 20° anteversion angle and a 40° abduction angle. (2) The lower edge of the acetabular cup is aligned with the teardrops’ line, with the cup size determined based on the anteroposterior diameter of acetabulum. (3) The inner edge of the acetabular cup is then gradually moved upwards along the Kohler line in the 2D view of AIHIP until the coverage of the cup reaches 70% in the 3D view. (4) Fine-tuning of eventual cup size is performed based on the actual situation and the surgeon’s preference. Implants in the database matched surgery with no offset. AI’s automatic placement can be adjusted by the operator intraoperatively if necessary.

### Preoperative planning with 2D templates

**Fig. 5:**
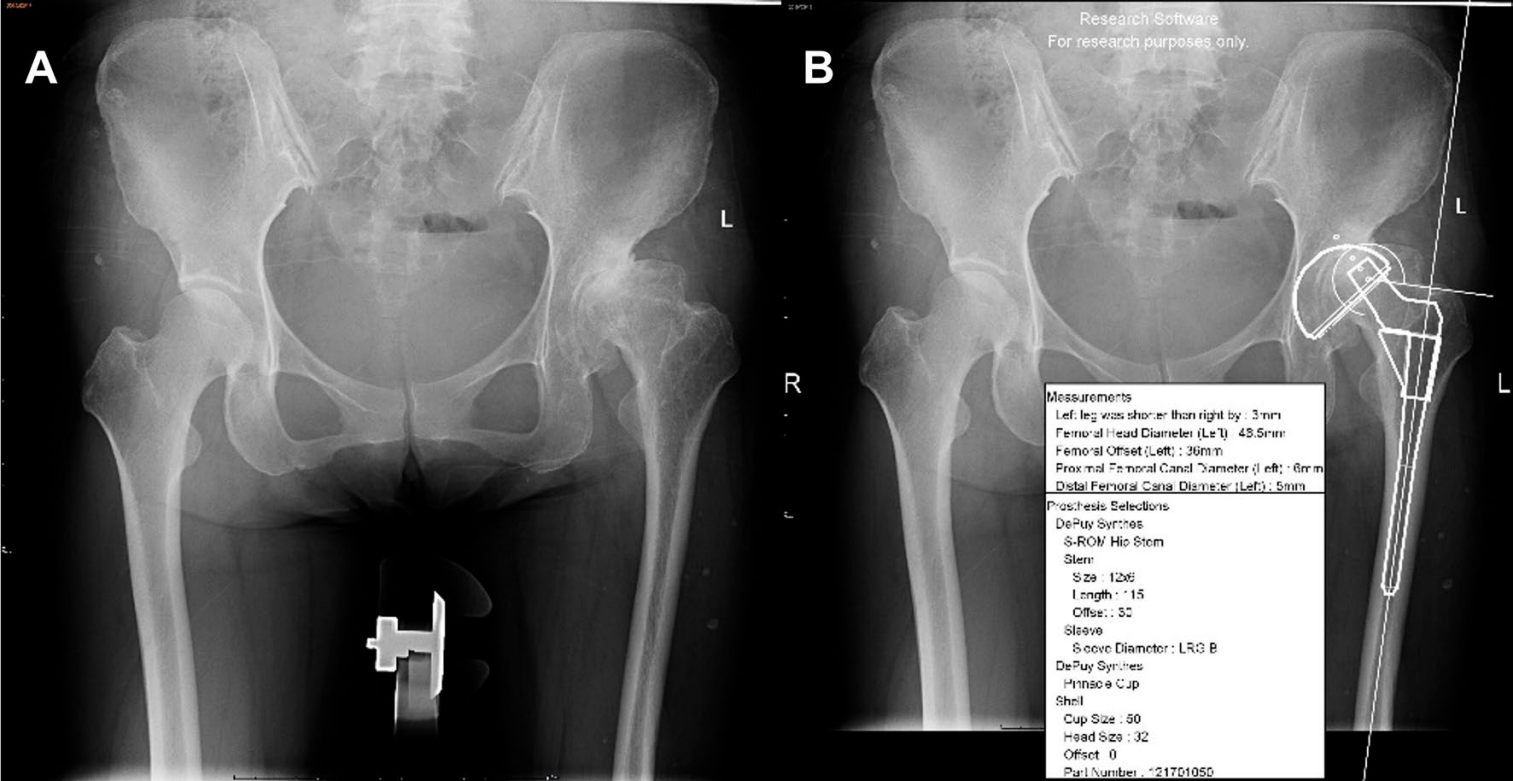
Schematic diagram of cup planning by a 2D template. **A** Standard preoperative anteroposterior X-ray of the pelvis. **B** Preoperative planning by the 2D template

Based on anteroposterior (AP) radiographs of the pelvis, a 2D digital template was performed in a standard manner using a 50 mm magnifying marker sphere in the perineal region at the level of the greater trochanter. A standard AP X-ray of the patient's pelvis was imported into the 2D digitizing template software OrthoView (Jacksonville, FL), and the diameter of the marker sphere was set to 50 mm. The software’s tools measured anatomical landmarks like the femoral head center and acetabular rim, helping select implant sizes and positioning. Templating selected implant sizes and orientation based on the patient's anatomy. For DDH, placement of the cup follows the same principles as AIHIP software: Crowe I and IV cups should be in anatomical position, while Crowe II and III cups should have at least 70% coverage and proper upward and medial movement. Finally, a report with implant size, orientation, and surgical notes was generated (Fig. 5).

### Surgical methods

The posterolateral approach was used for all patients, with a 15 cm incision along the posterior border of the greater trochanter. The capsule was resected, and the femoral head was removed. Hyperplastic tissue in the true acetabulum was cleaned, ground, and filed, gradually deepening it. The acetabular cup was placed in situ for Crowe I and IV DDH, while Crowe II and III cups had at least 70% coverage with appropriate upward and medial movement. The Pinnacle cup was inserted and the femoral head trialed before being implanted and reduced. Joint mobility was checked, and the incision was closed.

### Preoperative planning outcome assessment indicators

1. Coincidence rate [11, 14, 15]: The planned and actual sizes of the acetabular cup during surgery were compared to determine the coincidence rate. A "correct" coincidence was achieved if the planned and actual sizes were completely consistent. A planned size within ± 1 size was considered "accurate," while any size beyond that range was considered "inaccurate."
2. Average absolute error: The average absolute error was calculated as the average difference between the implanted and planned part sizes.

### Statistical methods

Statistical analysis was performed using SPSS 25.0 (IBM, United States) and GraphPad Prism version 8.0.2 (GraphPad Software, United States). The measurement data are expressed as the mean and standard deviation. If the data conformed to a normal distribution, intergroup comparisons were performed by independent sample *t* tests; otherwise, nonparametric tests were used. Enumeration data are expressed as frequency (number of cases, percentage), and the chi-square test was used for comparison between enumeration data. If the sample and frequency did not meet the requirements of the chi-square test, the chi-square value was corrected, or the probability was calculated by Fisher’s exact probability method. Test level *α* = 0.05 (two-sided).

The accuracy of preoperative planning was compared between the AIHIP and 2D template groups, and factors affecting accuracy were analyzed including gender, side, BMI (per Chinese standard: lean: < 18.5, normal: 18.5–23.9, overweight/preobesity: 24–27.9, obesity: ≥ 28) and DDH classification (Crowe I–IV). Detailed analysis of these factors influencing preoperative planning was conducted.

## Result

### Demographic information

A total of 103 patients (16 men, 87 women) with a mean age of 42.3 ± 12.4 years and a mean BMI of 24.0 ± 2.7 kg/m^2^ were studied. 164 hips were included, with 80 left and 84 right hips. The primary diagnosis was Crowe I (23 hips), Crowe II (38 hips), Crowe III (56 hips), and Crowe IV (47 hips). Patient information is in Table 1.

**Table 1:** General information of DDH patients undergoing THA

General information	Values
Age (years)	42.3 ± 12.4
Gender	*N* = 103
Male	16
Female	87
BMI (kg/m^2^)	24.0 ± 2.7
Side	*N* = 164
Left	80
Right	84
Classification of DDH	*N* = 164
Crowe I	23
Crowe II	38
Crowe III	56
Crowe IV	47

Data are expressed as the mean and standard deviation

### Accuracy of prosthesis size

**Fig. 6:**
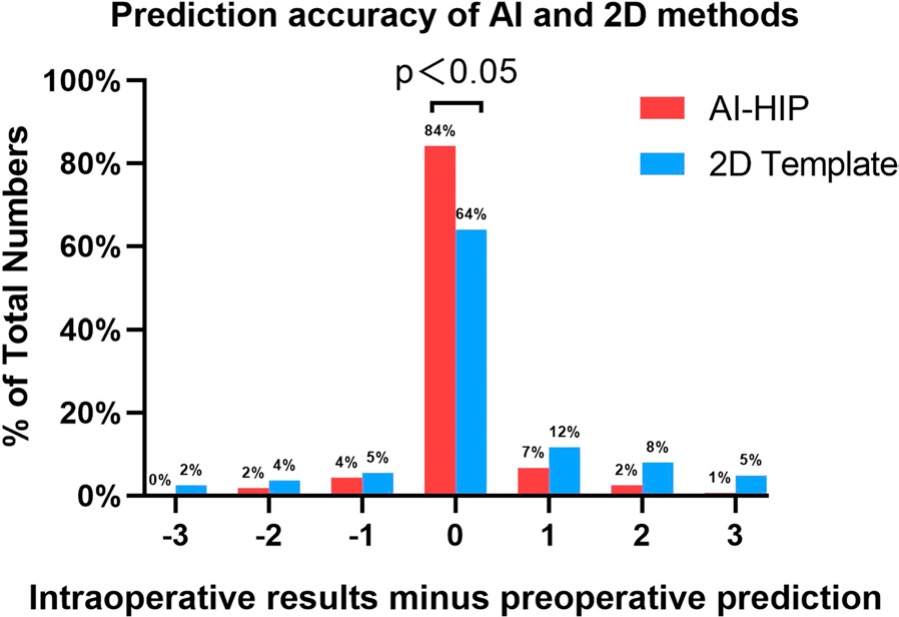
Prediction accuracy of Al and 2D methods

The AIHIP accurately predicted cup size within ± 0 and ± 1 size at 84.1% (138/164) and 95.1% (156/164), respectively. In comparison, the 2D template had accuracies of 64.0% (105/164) and 81.1% (133/164), respectively. The AIHIP was significantly more effective than the 2D template in templating the acetabular component size (*p* < 0.05) (see Fig. 6 and Table 2).

**Table 2:** Chi-square test for comparison of AIHIP and the 2D template for predicting cup size (within ± 1 size)

	Accurate	Inaccurate	*χ*^2^	*p*
*Method*				
AIHIP	156 (95.1%)	8 (4.9%)	15.395	0.000
2D template	133 (81.1%)	31 (18.9%)		

### Factors influencing the accuracy of AIHIP

As shown in Table 3, Chi-square testing of the factors affecting the accuracy of AIHIP preoperative planning revealed no statistically significant associated risk of gender, side, BMI and DDH classification on the accuracy of acetabular cup size planning in AIHIP (*p* > 0.05).

**Table 3:** Chi-square test for the accuracy of predicting cup of Al-HIP method (within ± 1 size)

Influencing factors	Subgroup	Accurate	Inaccurate	*n*	*χ*^2^	*p*
Gender	Female	142 (95.3%)	7 (4.7%)	149	0.000	1.000
	Male	14 (93.3%)	1 (6.7%)	15		
Side	Right	80 (95.2%)	4 (4.8%)	84	0.000	1.000
	Left	76 (95.0%)	4 (5.0%)	80		
BMI	< 18.5	6 (100.0%)	0 (0.0%)	6	3.984	0.236
	18.5–23.9	74 (94.9%)	4 (5.1%)	78		
	24.0–27.9	62 (93.9%)	4 (6.1%)	66		
	≥ 28	14 (100.0%)	0 (0.0%)	14		
DDH classification	Crowe I	23 (100.0%)	0 (0.0%)	23	6.144	0.060
	Crowe II	34 (89.5%)	4 (10.5%)	38		
	Crowe III	52 (92.9%)	4 (7.1%)	56		
	Crowe IV	47 (100.0%)	0 (0.0%)	47		

### Factors influencing the accuracy of the 2D template

In Table 4, chi-square testing indicated no statistically significant impact of gender, side, and BMI on the accuracy of acetabular cup size planning using the 2D template (*p* > 0.05), with no significant differences observed among them. However, a significant difference in accuracy was found among the four DDH classification groups (*p* = 0.000), indicating a significant impact of DDH classification on preoperative planning accuracy with the 2D template in DDH patients.

**Table 4:** Chi-square test for the accuracy of predicting the cup of the 2D template method (within ± 1 size)

Influencing factors	Subgroup	Accurate	Inaccurate	*n*	*χ*2	*p*
Gender	Female	121 (73.8%)	28 (17.1%)	149	0	1
	Male	12 (80.0%)	3 (20.0%)	15		
Side	Right	73 (86.9%)	11 (13.1%)	84	3.788	0.072
	Left	60 (75.0%)	20 (25.0%)	80		
BMI	< 18.5	6 (100.0%)	0 (0.0%)	6	1.357	0.726
	18.5–23.9	61 (78.2%)	17 (21.8%)	78		
	24.0–27.9	54 (81.8%)	12 (18.2%)	66		
	≥ 28	12 (85.7%)	2 (14.3%)	14		
DDH classification	Crowe I	23 (100.0%)	0(0.0%)	23	20.067	0
	Crowe II	26 (68.4%)	12 (31.6%)	38		
	Crowe III	38 (67.9%)	18 (32.1%)	56		
	Crowe IV	46 (97.9%)	1 (2.1%)	47		

The samples of the t test obey a normal distribution

Table 4 shows low accuracy of 2D template preoperative planning for Crowe II and III DDH. For further analysis, mean absolute error was used to evaluate the tendency to overestimate or underestimate component size. Table 5 and Fig. 6 demonstrate that, overall, the 2D template had a greater range of misestimation of cup size compared to AIHIP (*p* < 0.05) and was more likely to underestimate size. Comparison of mean absolute error in Table 5 revealed AIHIP to be significantly less than the 2D template for Crowe II and III (*p* < 0.05).

**Table 5:** T test for mean absolute error between postoperative outcome and preoperative planning of cup size

		AIHIP (*n* = 164)	2D Template (*n* = 164)	*t* value	*p* value
Total (*n* = 164)		0.21 ± 0.54	0.62 ± 0.95	− 4.821	0
DDH					
Classification	Crowe I (*n* = 23)	0.04 ± 0.21	0.17 ± 0.38	− 1.421	0.162
	Crowe II (*n* = 38)	0.32 ± 0.66	0.97 ± 1.15	− 3.055	0.003
	Crowe III (*n* = 56)	0.36 ± 0.67	0.98 ± 1.05	− 3.594	0.001
	Crowe IV (*n* = 47)	0.04 ± 0.20	0.13 ± 0.40	− 1.308	0.194

### Analysis of typical cases

**Fig. 7:**
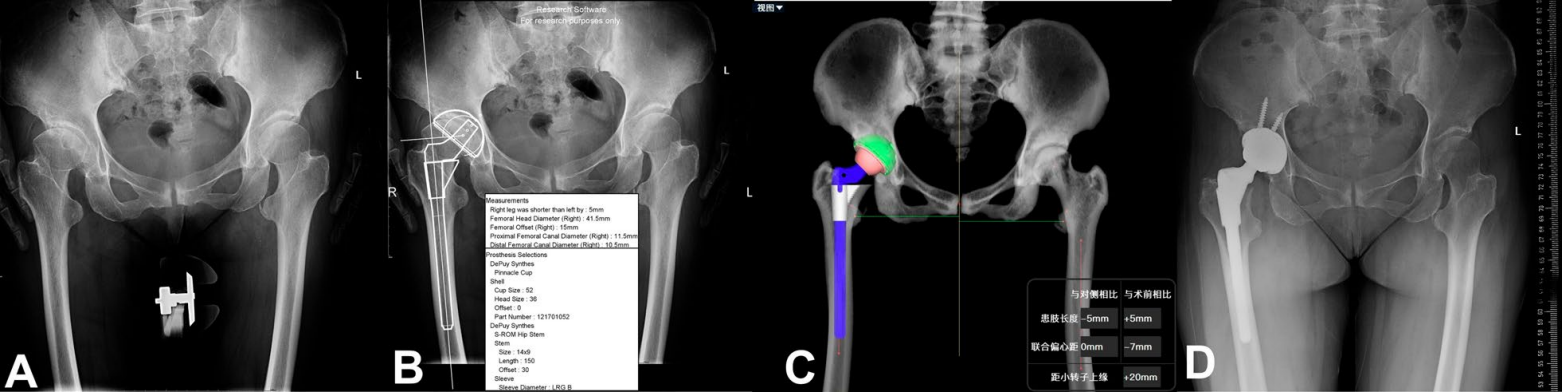
Case 1, woman, 54 years old, Crowe I DDH, left. **A** Standard preoperative anteroposterior X-ray of the pelvis. **B** Preoperative planning by the 2D template; planning results: Cup size was 50 mm. **C** Preoperative planning by AIHIP; planning results: Cup size was 50 mm. **D** Standard postoperative anteroposterior X-ray of the pelvis: Cup size was 50 mm

**Fig. 8:**
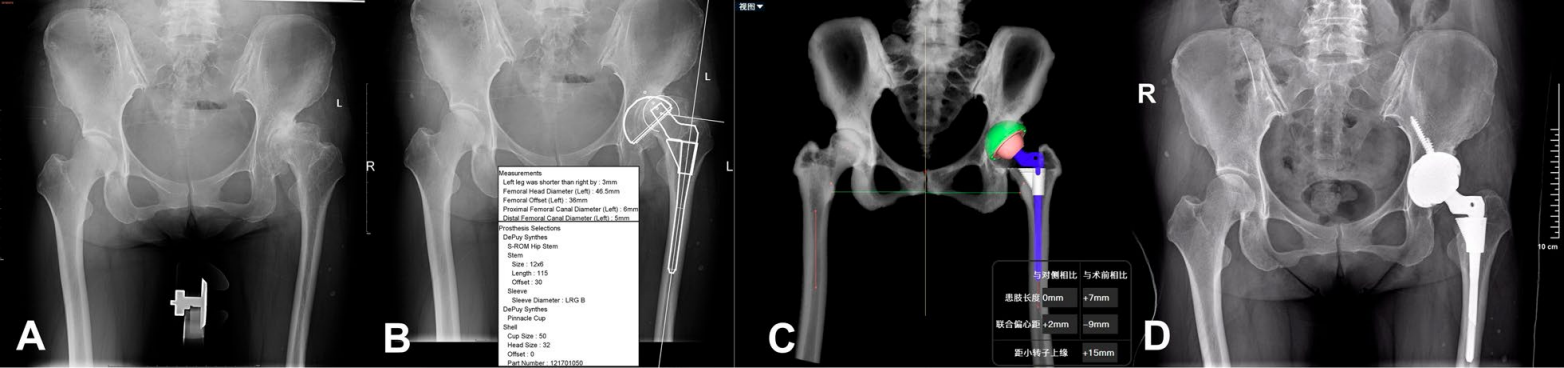
Case 2, woman, 51 years old, Crowe II DDH, left. **A** Standard preoperative anteroposterior X-ray of the pelvis. **B** Preoperative planning by the 2D template; planning results: Cup size was 50 mm. **C** Preoperative planning by AIHIP; planning results: Cup size was 52 mm. **D** Standard postoperative anteroposterior X-ray of the pelvis: Cup size was 52 mm

**Fig. 9:**
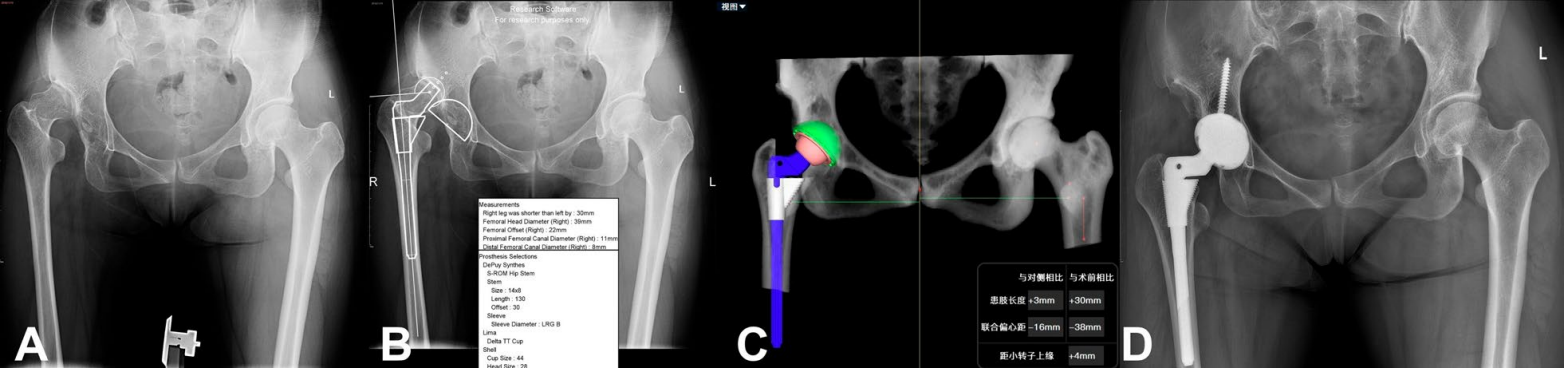
Case 3, woman, 47 years old, Crowe III DDH, right. **A** Standard preoperative anteroposterior X-ray of the pelvis. **B** Preoperative planning by the 2D template; planning results: Cup size was 44 mm (Since the OrthoView software does not have a 44 mm Pinnacle cup, a 44 mm Lima cup was planned by the 2D template preoperatively, but the Pinnacle cup was used intraoperatively). **C** Preoperative planning by AIHIP; planning results: Cup size was 46 mm. **D** Standard postoperative anteroposterior X-ray of the pelvis: Cup size was 46 mm

**Fig. 10:**
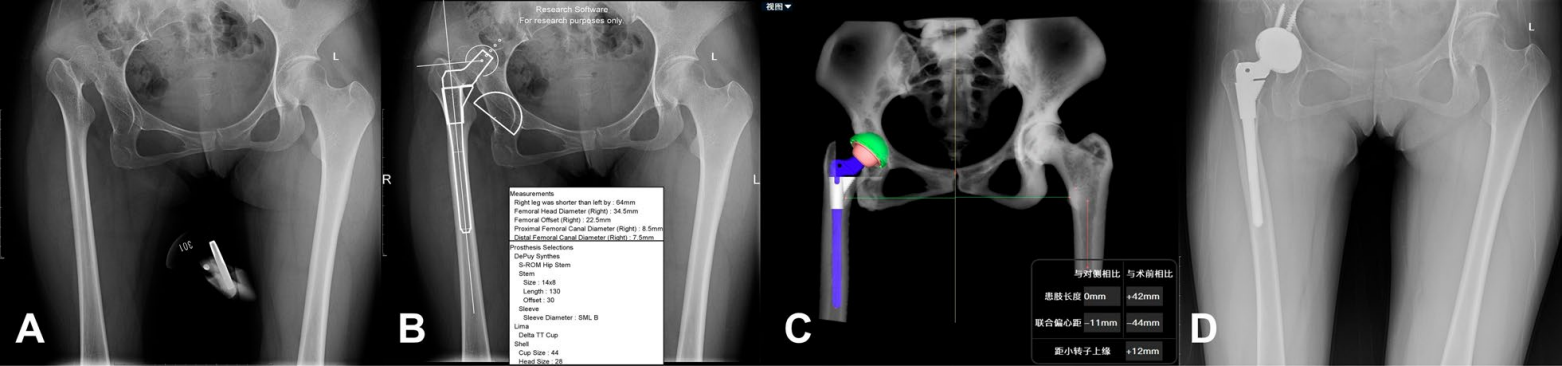
Case 4, woman, 35 years old, Crowe IV DDH, right. **A** Standard preoperative anteroposterior X-ray of the pelvis. **B** Preoperative planning by the 2D template; planning results: Cup size was 44 mm (Since the OrthoView software does not have a 44 mm Pinnacle cup, a 44 mm Lima cup was planned by the 2D template preoperatively, but the Pinnacle cup was used intraoperatively. **C** Preoperative planning by AIHIP; planning results: Cup size was 44 mm. **D** Standard postoperative anteroposterior X-ray of the pelvis: Cup size was 44 mm

Based on the AI-assisted preoperative planning method AIHIP, we analyzed four cases of DDH with varying severity. We compared the accuracy of the 3D AIHIP plan with the 2D template plan and actual surgery outcome for each case. (see Figs. 7, 8, 9 and 10).

AIHIP is valuable in preoperative planning for THA in DDH patients, especially in cases where 2D templates may fail due to complex anatomy and DDH classification. These cases demonstrate that AIHIP can provide accurate 3D planning, leading to good surgical outcomes and increasing the success rate of THA in DDH patients.

## Discussion

THA requires accurate preoperative planning of acetabular cup size to reduce dislocation risk, increase joint stability, and improve surgical success. This study investigated the value of AIHIP in THA for patients with DDH. AIHIP was more accurate than 2D templates for preoperative planning of cup size in DDH patients and was unaffected by age, gender, side, or BMI. AIHIP was also accurate for different classifications of DDH, whereas the 2D template was less accurate for Crowe II and Crowe III patients. AIHIP effectively solved measurement errors caused by DDH classification for the 2D template. In conclusion, AI-assisted 3D planning accurately predicts cup size in DDH arthroplasty and is particularly useful in cases with limited accuracy using the 2D template, such as Crowe II and Crowe III DDH.

The accuracy of preoperative planning for acetabular cup size has also been reported in different studies. According to previous studies, the accuracy of cup size matching within ± 1 also ranged from 59 to 77% in studies with 2D template analysis [11, 12, 16, 17]. In studies analyzed manually with 3D templates, the accuracy of cup size matching within ± 1 also varied from 90 to 93% [11, 18]. In the AIHIP software used in this study, the accuracy of acetabular cup size matching within ± 1 also ranged from 94 to 97% [6, 11, 12]. The accuracy reported in this study falls between these reports. Although CT-based 3D THA template software has higher accuracy than traditional 2D templates [18], it is also more time-consuming [11]. The AIHIP software used in this study can greatly save time compared with the 3D template [6, 11, 12] because it does not require manual planning and has higher accuracy than the two-dimensional template; consequently, it has certain advantages in clinical application. However, there is no corresponding report on the associated risk of different hip development classifications on preoperative planning with the AIHIP and 2D templates. The innovation of this experimental design is that different types of DDH were selected as the research objective, and the preoperative planning results of AIHIP and the 2D template were compared. The 2D template also has a high accuracy rate for patients with common hip arthritis and femoral head necrosis; however, patients with DDH have complex conditions and abnormal anatomical structures, and it is difficult to perform accurate preoperative planning with the common 2D template. In this study, although AIHIP had higher precision than the 2D template for each type of DDH, the reason why AIHIP had a significantly different error in predicting acetabular cup size from the 2D template for Crowe II and Crowe III DDH is as follows: For DDH patients with Crowe II and III, the acetabular cup position can be challenging to control due to dysplasia, which affects the rasping of the bone bed. Surgeons often increase the depth of acetabular bottom rasping to increase bony contact, resulting in higher errors between the preoperative plan and intraoperative size.

This study has several limitations: 1. It only analyzed the size of the acetabular cup in DDH; all acetabular cups were Pinnacle acetabular cup prostheses, so our study conclusion is limited. 2. We did not consider the size of the femoral component because there are many types of femoral components used clinically, especially modular prosthesis stems, with various combinations of neck and head, and it is difficult to clarify the repeatability of this modularization. Therefore, further detailed studies are needed to verify the reliability of preoperative AIHIP for different types of femoral components. 3. Although the study subjects were young patients with DDH, some patients still had osteoporosis symptoms during the operation. Even if the size of the acetabular cup was appropriate, the clamping force was not sufficient, and the surgeon was forced to increase the size to ensure the stability of the acetabular cup during the operation. This had an impact on the results of the study. 4. For DDH patients with Crowe II and Crowe III hips, there are no consistent criteria for superior and internal displacement of the cup in artificial hip arthroplasty surgery, and this lack of consistency may have an impact on the accuracy of the study results. 5. Our study is retrospective, with strict inclusion criteria, and it is difficult to obtain data by blinding and to follow the random principle. Without the prior intervention of the investigator, the study results are inevitably biased. In a follow-up investigation, we will continue to increase the sample size and upgrade the machine learning to achieve personalized matching between the operator's surgical concept and the patient.

## Conclusion

The AI-assisted 3D planning system AIHIP is more accurate than traditional 2D templates and can accurately predict the cup size for DDH joint arthroplasty. Crowe II and Crowe III typing in DDH patients is a factor contributing to the 2D template, whereas the use of AIHIP can avoid this effect.

## Data Availability

No datasets were generated or analyzed during the current study.
